# The ferroptosis signature predicts the prognosis and immune microenvironment of nasopharyngeal carcinoma

**DOI:** 10.1038/s41598-023-28897-2

**Published:** 2023-02-02

**Authors:** Ji Zhou, Tianyao Guo, Lin Zhou, Meihua Bao, Lin Wang, Wenhu Zhou, Shan Tan, Guangyi Li, Binsheng He, Zhen Guo

**Affiliations:** 1 Academician Workstation, Changsha Medical University, Changsha, China; 2 Department of Pathology, The Second Xiangya Hospital of Central South University, Changsha, China; 3 Department of Emergency Medicine, The First Affiliated Hospital of Changsha Medical University, Changsha, China

**Keywords:** Cancer, Head and neck cancer

## Abstract

Nasopharyngeal carcinoma (NPC) is a cancer with a high metastatic rate and poor prognosis. Growing studies suggest that ferroptosis take part in the development of tumours. At the same time, the connection between ferroptosis-related genes (FRGs) and the prognosis of NPC remains unclear. In this study, we explored the dysregulated FRGs between normal control and tumour samples of NPC. Firstly, 14 of 36 differentially expressed FRGs were identified in NPC tissues compared to normal tissues, among which ABCC1, GLS2, CS and HMGCR were associated with poor prognosis for patients. The four ferroptosis genes were used for consensus cluster analysis and two risk-related FRGs (ABCC1 and GLS2) were used in a risk model. The ROC curve revealed the good predictive performance of this risk signature. Multivariate analysis revealed that risk score and intratumoral TILs were independent risk factors linked to prognosis. Additionally, our results suggested that the risk signature was attached to the immune microenvironment. Moreover, the NPC patients with high risk were sensitive to chemotherapeutic drugs including axitinib, docetaxel, embelin, epothilone.B, parthenolide, thapsigargin, tipifarnib, vinorelbine. Finally, the expression of ABCC1 and GLS2 was validated in NPC tissues using immunohistochemistry. Together, these results revealed ferroptosis may be a potential biomarker in NPC and representing a promising future direction in prognosis and therapeutic strategy for the treatment of NPC.

## Introduction

Nasopharyngeal carcinoma (NPC) is a cancer with a poor prognosis and high metastasis rate that frequently occurs in southern China^1,2^. Every year, there are over 133,000 new cases, resulting in 80,000 deaths from NPC in 2020^3^. The incidence of NPC has been increasing annually. Epstein‒Barr virus (EBV) infection, preserved foods and alcohol consumption are important risk factors for developing NPC^4,5^. Despite the availability of conventional treatments, such as surgery, radiotherapy and chemotherapy, the risk of recurrence and metastasis remains high^6^. Therefore, it is vital to understand its pathogenesis and identify novel and reliable targets related to prognostic risk stratification for treating NPC.

Ferroptosis is a new cell death mode, characterized as an oxidative cell death induced by small molecules in certain circumstances, dependent on iron ions^7^. Ferroptosis is caused by an imbalance in the generation and degradation of intracellular reactive oxygen species (ROS)^8^. Several studies have confirmed that FRGs are associated with the clinical diagnosis, prognosis and progression of various cancers, including breast carcinoma, colorectal carcinoma, lung adenocarcinoma and glioma^9–11^. ABCC1 is involved in tumor progression, drug resistance and poor patient survival in classical Hodgkin lymphoma (CHL)^12^. GLS2 participates in p53-mediated ferroptosis and is related to hepatocellular carcinoma, cervical carcinoma and lung adenocarcinoma. Given the high iron levels of cancer cells and their ferroptosis sensitivity, ferroptosis has been proposed to be promising for tumor therapeutics^13^.

The tumor microenvironment (TME) is a unique internal environment that includes macrophages, stromal cells, tumor-infiltrating lymphocytes, and some immune cells^14^. It plays a vital role in tumorigenesis and therapeutic strategies^15,16^. Immunotherapy is a recently developed modality for treating recurrent or metastatic disease and has excellent clinical value^17^. Recent studies have shown cooperation between tumor ferroptosis and the TME. Wang et al. found that CD8 + T cells participate in antitumor activity by promoting tumor ferroptosis, which may be a potential therapeutic strategy^18,19^. However, the relationship between ferroptosis and the immune microenvironment/immunotherapy of NPC patients is unclear.

In this study, we comprehensively explored the expression and prognosis of FRGs from 3 NPC Gene Expression Omnibus (GEO) datasets and designed a risk signature containing 2 FRGs. Next, we researched the communication between the FRG risk signature and immune infiltration. We then investigated the correlation between the risk score and drug sensitivity for NPC therapy. Finally, the expression of ABCC1 and GLS2 was validated in tissues using immunohistochemistry (IHC).

## Results

### FRGs related to survival in NPC

**Figure 1:**
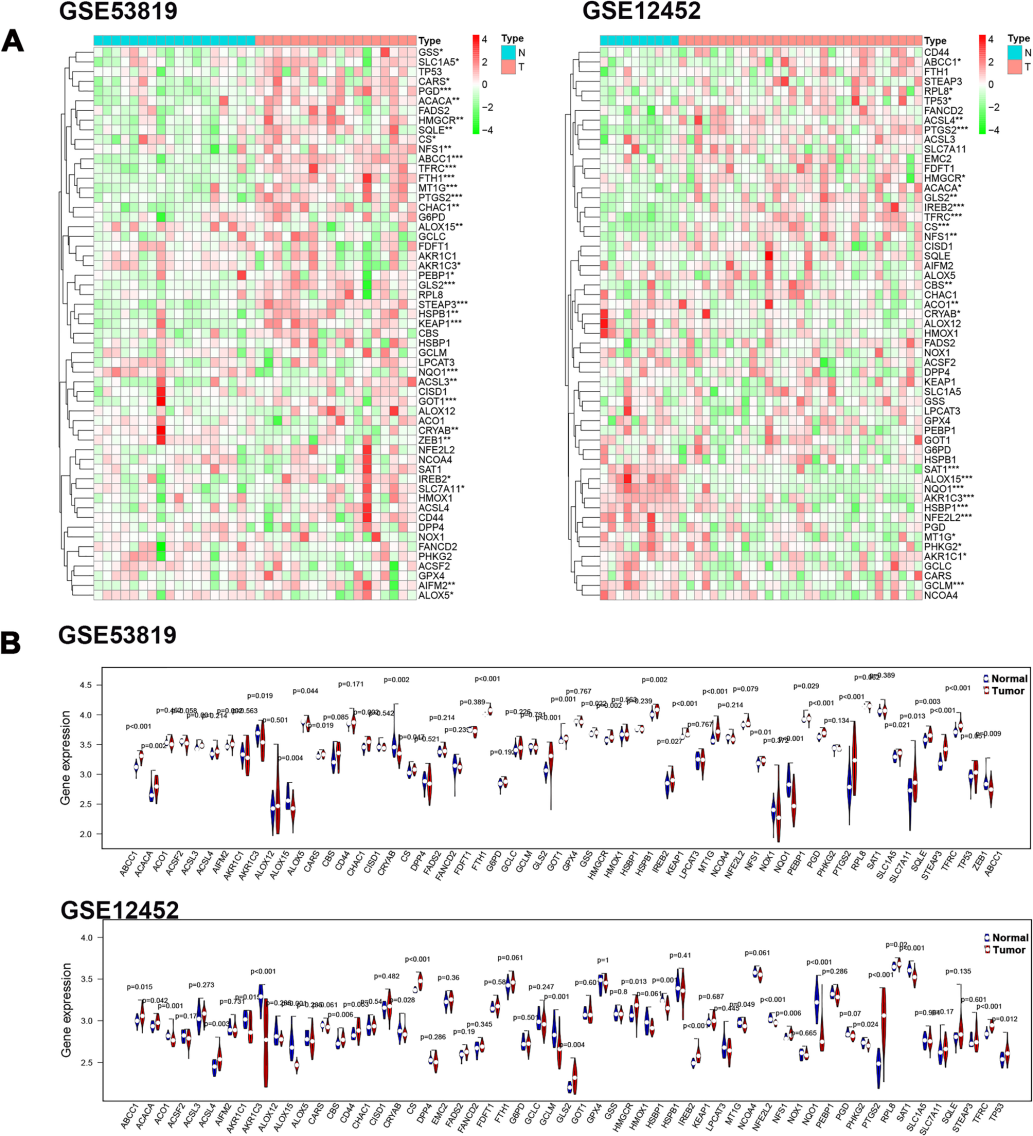
The expression profiles of FRGs in NPC. (**A**) The heatmap of DE_FRGs in GSE53819 and GSE12452 using “edgeR” package (version 4.0.5, https://cran.r-project.org/). (**B**) The gene expression levels of FRGs in GSE53819 and GSE12452 from GEO (https://www.ncbi.nlm.nih.gov/geo/).

**Figure 2:**
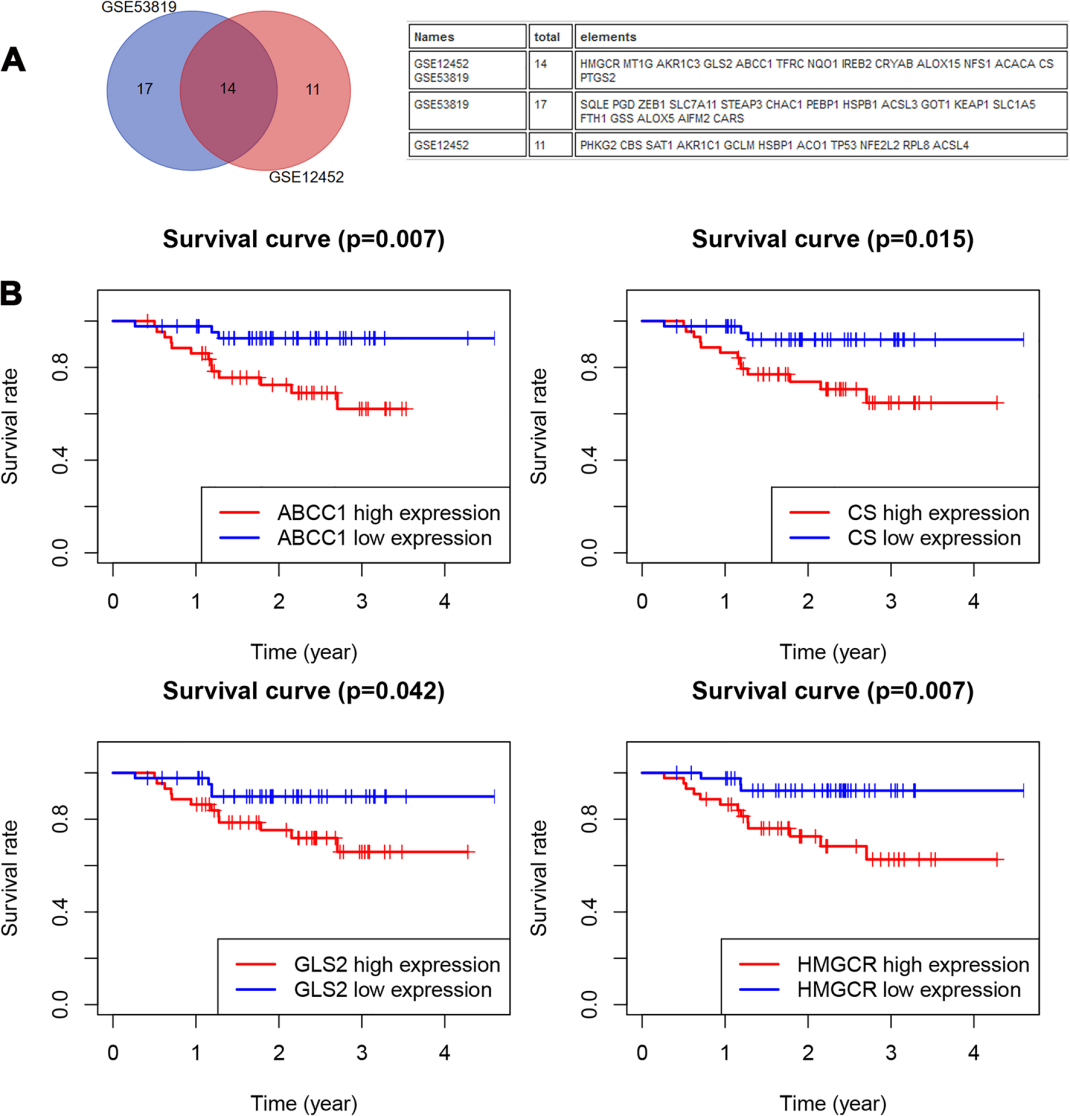
The expression of FRGs in NPC. (**A**) The overlapped DE_FRGs from GSE53819 and GSE12452. (**B**) K–M curves of poor progression-free survival for ABCC1, CS, GLS2 and HMGCR expression in GSE12452.

To investigate the regulation of FRGs, we downloaded GSE53819 and GSE12452 from the GEO database. As shown in Fig. 1A, 25 differentially expressed FRG genes (DE_FRGs) were observed in GSE12452. In addition, 31 DE_FRGs were obtained in GSE53819 (Fig. 1B). Moreover, 14 DE_FRGs overlapped in both GSE12452 and GSE53819 (Fig. 2A). Next, we analyzed the potential prognostic role of 14 FRGs in GSE102349. We determined that 4 FRGs (ABCC1, CS, GLS2 and HMGCR) were associated with poor PFS in NPC (Fig. 2B).

### Estimation of consensus clustering of ferroptosis genes

**Figure 3:**
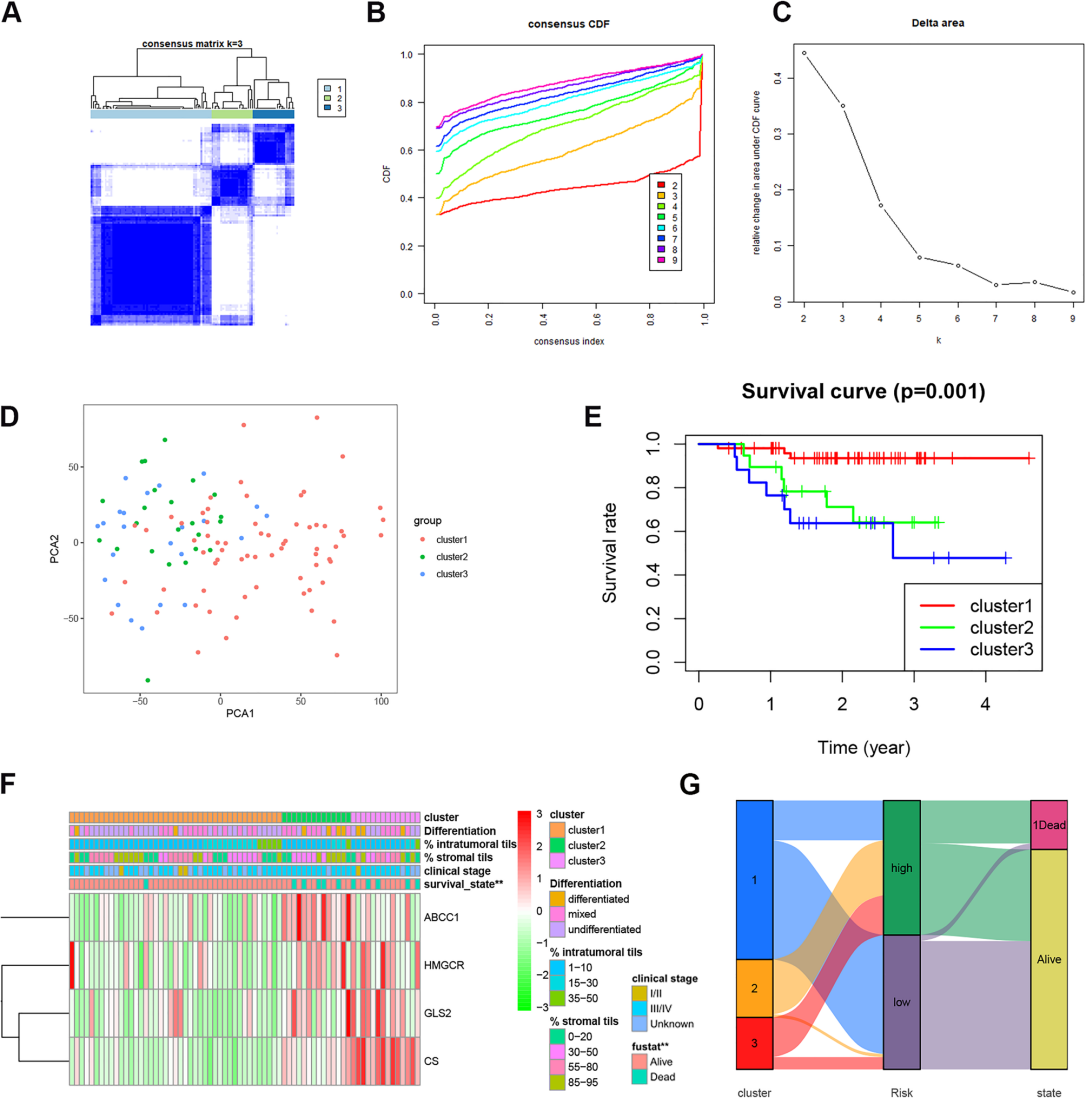
Consensus Clustering of FRGs of NPC. (**A**) Consensus clustering analysis using the R package “ConsensusClusterPlus” (https://cran.r-project.org/); (**B,C**) Consensus clustering cumulative distribution function; (**D**) PCA analysis for three clusters; (**E**) K-M curves of poor progression-free survival for three clusters; (**F**) The heatmap show the four clusters’ clinical characteristics of FRG. (**G**) Correlation analysis of clustering and risk classification using the R package “ggalluvial” and “ggplot2” (https://cran.r-project.org/).

Next, consensus clustering was performed to analyze four prognosis-related ferroptosis genes (ABCC1, HMGCR, CS and GLS2), and k = 3 may be the best choice in the GSE102349 datasets (Fig. 3A–C). The PCA and Kaplan‒Meier curve analysis of poor progression-free survival suggested that three clusters could be distinguished (Fig. 3D,E). The heatmap revealed that HMGCR and GLS2 were upregulated in the Cluster 2 and 3 subgroups, ABCC1 was upregulated in the Cluster 2 subgroup, and CS was upregulated in the Cluster 3 subgroup (Fig. 3F). Moreover, the correlation analysis of clustering and risk classification was performed using ggalluvial (Fig. 3G), and the results revealed that most patients in Cluster 2 and Cluster 3 were grouped into the high-risk group and were associated with a high proportion of NPC deaths. These results indicated that the expression of ferroptosis genes was associated with cluster subgroups and clinical characteristics.

### Prognostic risk model of FRGs in NPC

**Figure 4:**
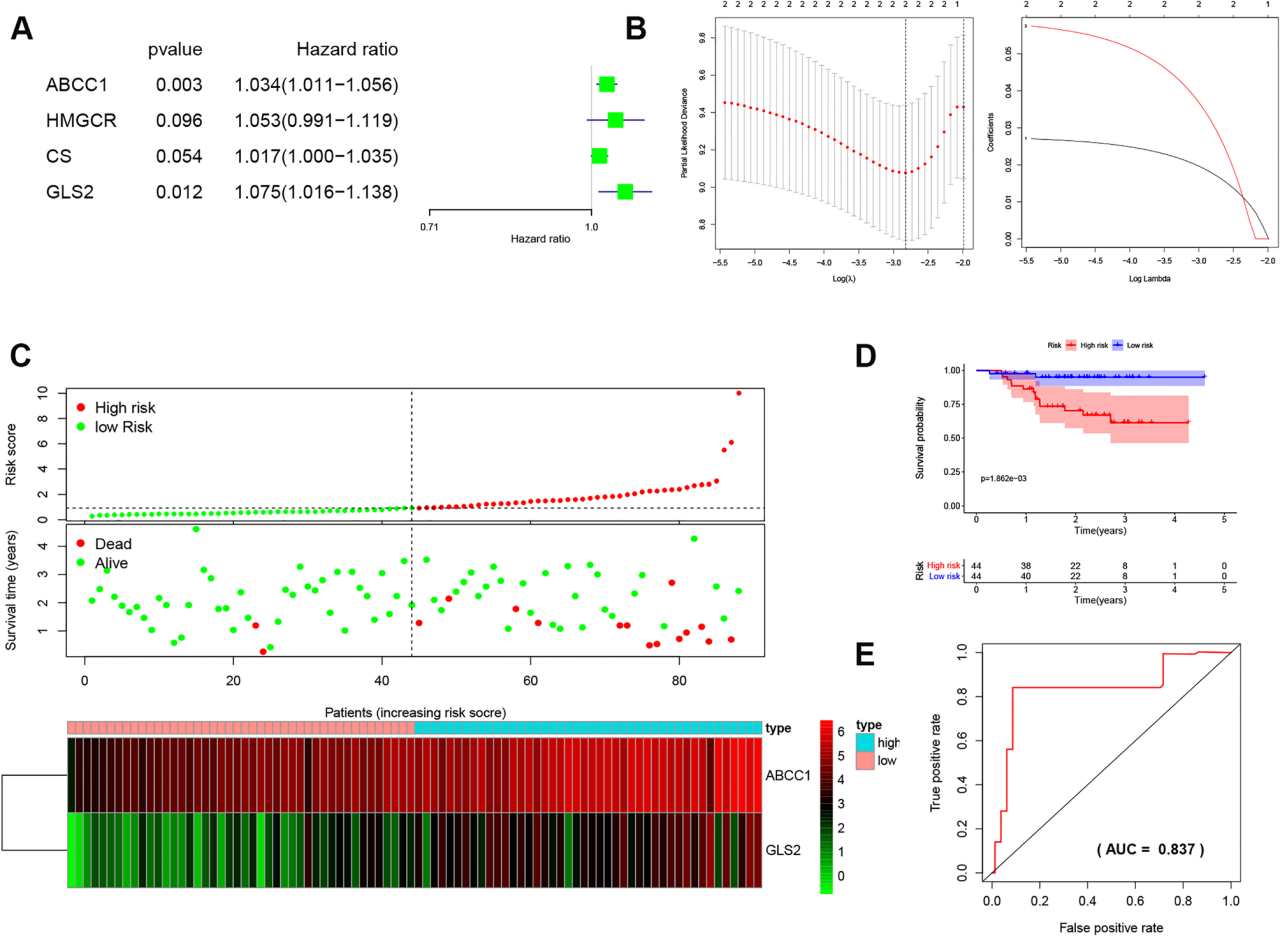
Risk model from three FRGs. (**A**) Univariate Cox analysis between four FRGs and poor progression-free survival of NPC using the R package “ConsensusClusterPlus”. (**B**) LASSO calculated the risk model using the R package “glmnet” (https://cran.r-project.org/src/contrib/Archive/glmnet/). (**C**) The distributions from two prognostic FRGs. (**D**) Kaplan–Meier poor progression-free survival curves for patients in risk score. (**E**) ROC curve showed the efficiency of the risk score model using the R package “survivalROC”.

Next, LASSO and Cox regression analyses were performed to identify an FRG signature for predicting the prognosis of NPC. As shown in Fig. 4A and B, two FRGs (ABCC1 and GLS2) were used to establish the risk model, and the risk score was ABCC1 × 0.02775 + GLS2 × 0.0592. The distributions of risk scores, survival times and expression profiles were based on the two FRGs (Fig. 4C,D). The ROC curve analysis showed that the area under the curve (AUC) of 3-year OS was 0.837 (Fig. 4E). The results indicated that the risk score model exhibited stable performance.

### Prognostic signature of FRGs in NPC

**Figure 5:**
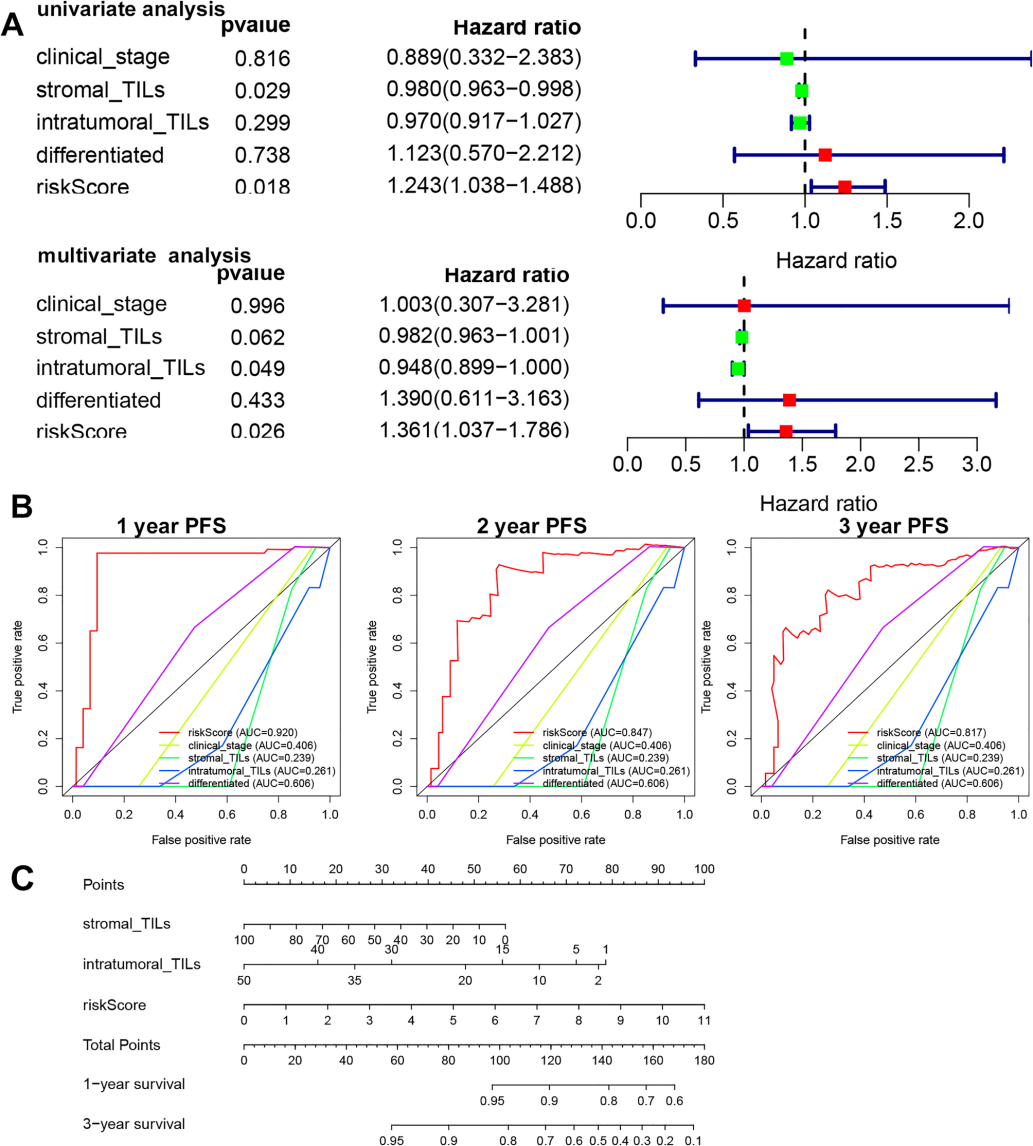
The independent predictive analysis. (**A**) COX regression analyses to identify prognosis signature. (**B**) The ROCs analysis to detect the predictive ability of clinical parameters. (**C**) Establishment of the poor progression-free survival nomogram for NPC patients.

To explore the independent prognostic signature in NPC, we evaluated the predictive value of clinical stage, stromal TILs, intratumoral TILs, differentiation and risk scores by performing univariate and multivariate analyses. Univariate analysis showed that stromal TILs and the risk score were significantly associated with progression-free survival (PFS). Multivariate Cox analysis showed that the risk score and intratumoral TILs were independent prognostic signatures (Fig. 5A). The ROC analysis showed that the AUC values were 0.92, 0.847, and 0.817 for 1-, 2-, and 3-year OS, respectively (Fig. 5B). Then, we constructed a nomogram to integrate risk scores, stromal TILs and intratumoral TILs, which can predict the probability of survival for NPC patients (Fig. 5C).

### The correlation between risk score and clinical features

**Figure 6:**
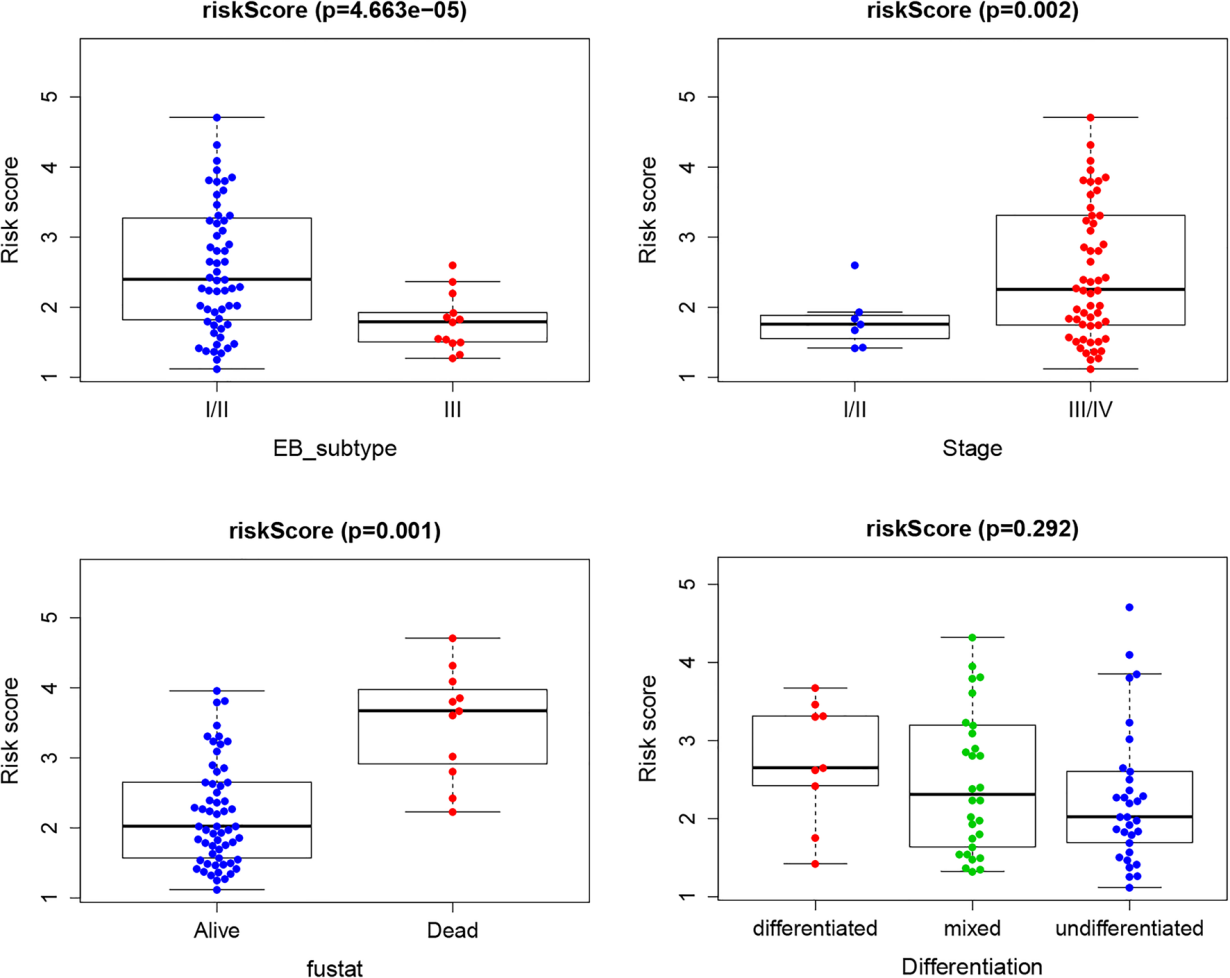
The correlation between risk score and clinical features. The risk score was linked to EB subtype, tumour grade, clinical stage, survival status and differentiation.

To reveal the communication between the risk score and clinical data of NPC, the correlation between clinical features (including the expression-based (EB) subtype, stage, survival status and differentiation) and the risk score was studied. The results revealed that patients with EB subtype I/II had a higher risk scores than those with subtype III, while patients in the advanced stage (III/IV) had a higher risk scores than those in the early stage (I/II). The risk score in deceased patients was higher than that in living patients. In contrast, the risk score between the differentiated and undifferentiated groups showed no significant differences. Together, the results indicated that the risk score was related to EB subtype, clinical stage and survival status (Fig. 6).

### Risk-related signaling pathways

**Figure 7:**
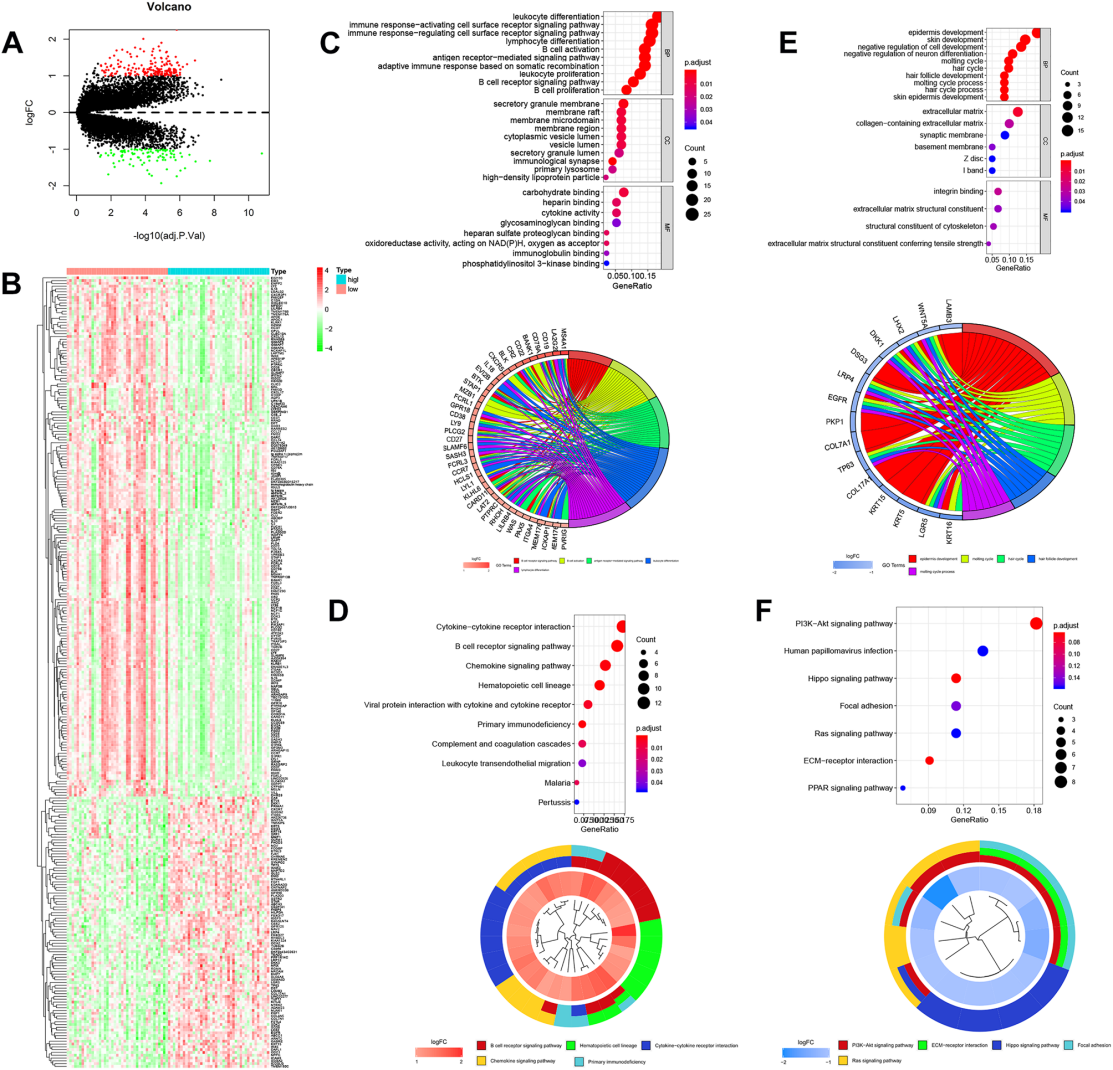
The risk status of DEGs. (**A**) The volcano map from DEGs with different risk status. (**B**) The DEGs heatmap form the different risk-status. The functional enrichment of DEGs, including GO (**C,E**) and KEGG (**D,F**) analysis using R packages “clusterProfiler” and “enrichplot”.

To investigate the potential functions of DEGs in risk-related NPC, a total of 201 upregulated (red) and 86 downregulated (green) DEGs were identified in the high-risk group (Fig. 7A). The heatmap of 287 DEGs was identified between high/low risk (Fig. 7B). The GO functional analysis suggested that the DEGs are mainly related to immune cell development, including leukocyte differentiation, lymphocyte differentiation, B-cell activation, lymphocyte proliferation, and B-cell proliferation (Fig. 7C,E). The KEGG functional enrichment analysis indicated that the DEGs were enriched in the B-cell receptor signaling pathway, PI3K-Akt signaling pathway, primary immunodeficiency pathway, Ras signaling pathway, and PPAR signaling pathway (Fig. 7D,F).

### Relationship of the risk score with immune infiltration in NPC

**Figure 8:**
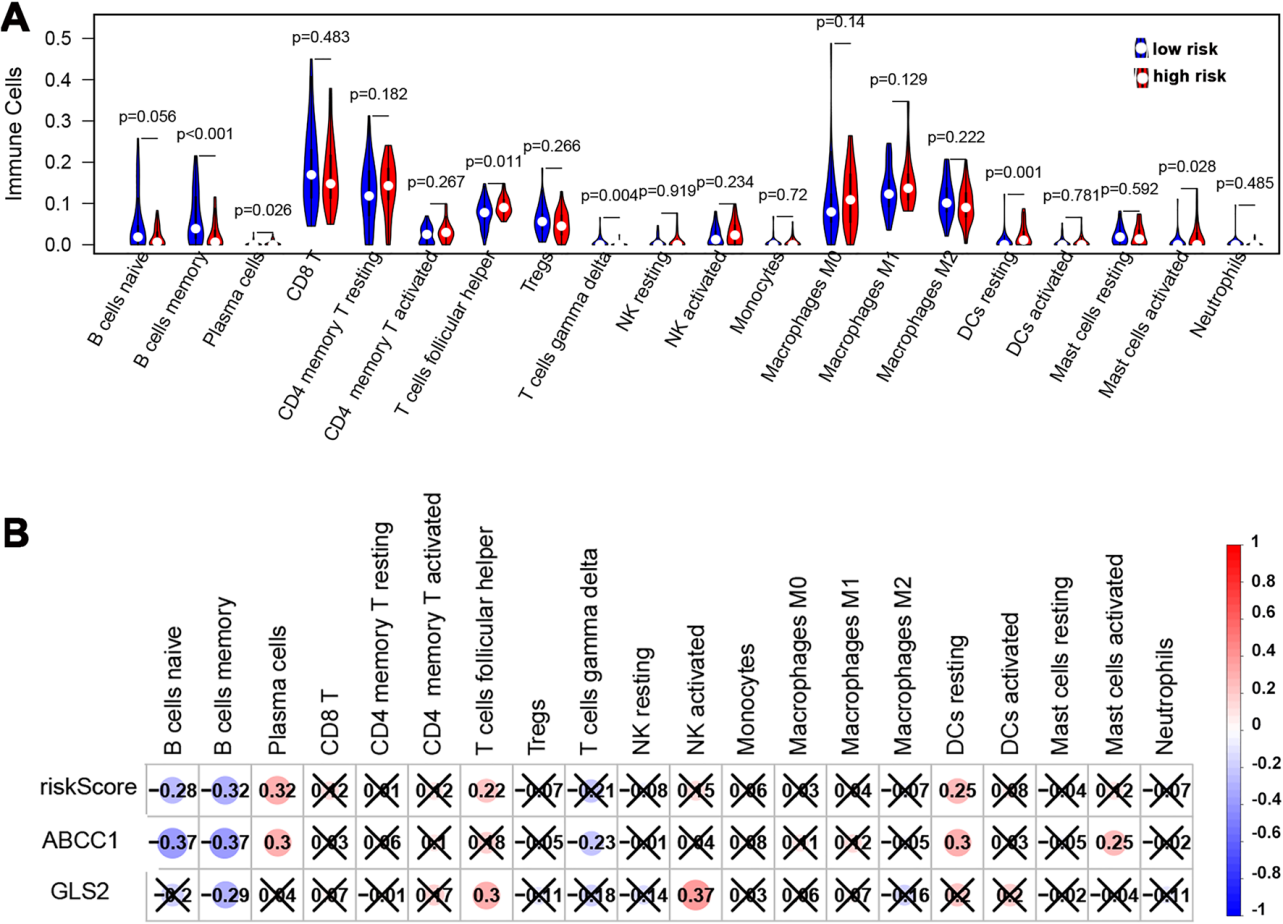
Immune infiltration in different risk-status. (**A**) The immune cell infiltration in the high/low-risk patients. (**B**) The risk score, FRGs ABCC1 and GLS2 were linked to infiltration abundances in different immune cells.

We used the CIBERSORT method to investigate immune infiltration in NPC patients in the low- and high-risk groups. The results revealed different immune infiltration in the high- and low-risk groups, including resting DCs and follicular helper T cells, while higher infiltration scores of naive B cells and memory B cells were observed in the low-risk patients (Fig. 8A). Then, we analyzed the relationship between the risk score and the infiltration of several immune cells (Fig. 8B). The results suggested that the risk score was significantly positively correlated with plasma cells, follicular helper T cells, and resting DCs but negatively associated with memory B cells and naive B cells. Moreover, ABCC1 was positively associated with plasma cells, follicular helper T cells, resting DCs, and resting mast cells but negatively associated with memory B cells, naive B cells, and gamma delta T cells. GLS2 was strongly positively correlated with activated NK and follicular helper T cells, while it was negatively correlated with memory B cells. These results revealed that the ABCC1 and GLS2 prognostic signatures for NPC patients are associated with immune infiltration.

### The relationship between risk score and chemotherapeutic drugs

**Figure 9:**
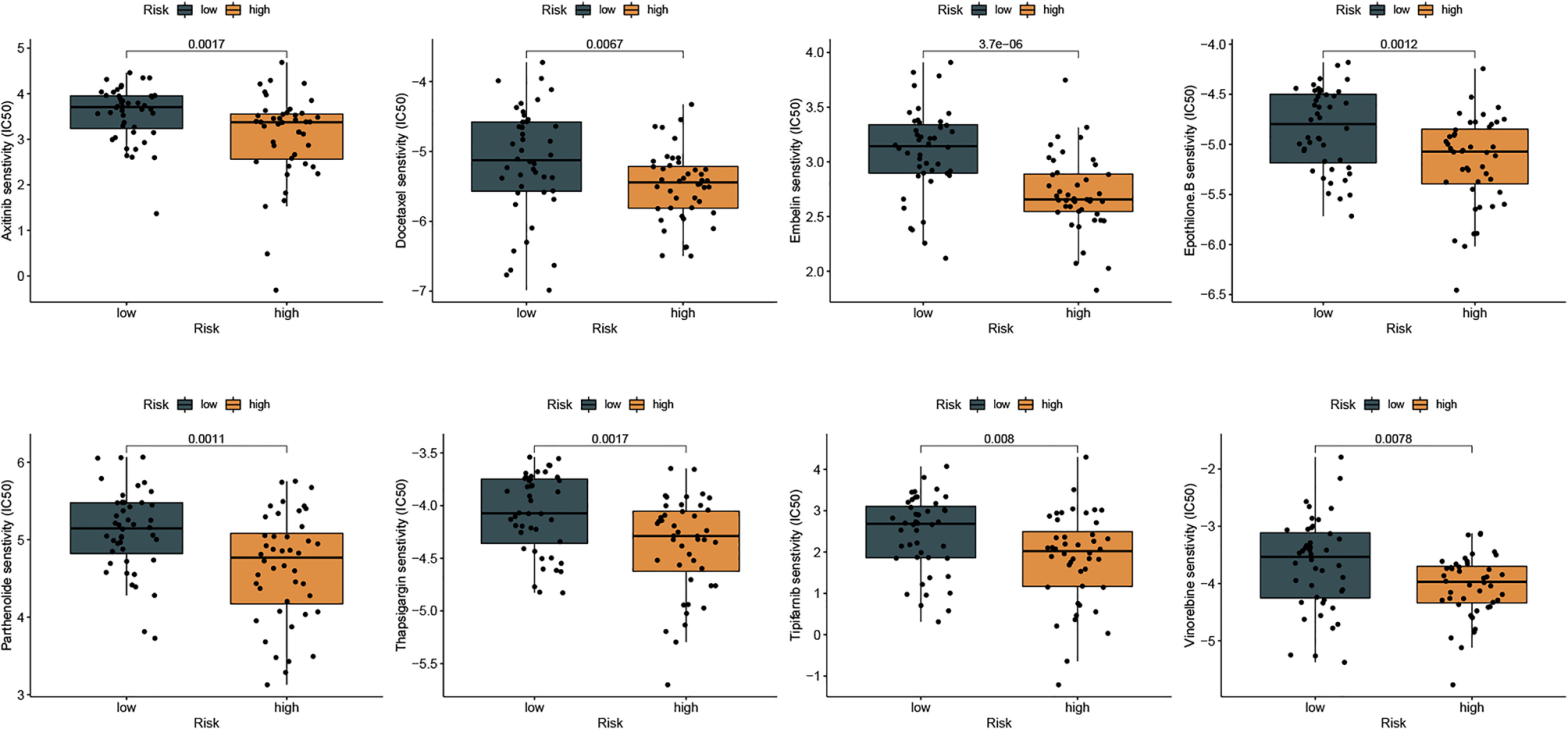
The relationships between low/high-risk groups and chemotherapeutics. Drugs testing is assessed by IC50, such as Axitinib, Docetaxel, Embelin, Epothilone.B, Parthenolide, Thapsigargin, Tipifarnib, Vinorelbine.

To select effective anticancer drugs for NPC patients, we explored the relationships between the risk score and chemotherapeutic agents. Our study showed that high-risk NPC patients were more sensitive to agents including docetaxel, embelin, and epothilone B, parthenolide, thapsigargin, tipifarnib, axitinib, and vinorelbine, suggesting that the risk scores may be a potential indicator of chemical sensitivity in the treatment of NPC (Fig. 9).

### Validation of the expression and prognosis of ABCC1 and GLS2 in NPC

We analyzed the expression of ABCC1 and GLS2 in pancancers using GEPIA2 (Figs. S1, S2). The results demonstrated that the expression levels of ABCC1 and GLS2 were diverse in various types of tumors. Since there is no NPC data in The Cancer Genome Atlas (TCGA), HNSC data were used to evaluate NPC in some articles^20–22^. Here, we found that ABCC1 was overexpressed in HNSC, which was consistent with the expression of ABCC1 in NPC. Nevertheless, contrary to our results, GLS2 expression was decreased in HNSC. Given that HNSC data are not exclusively derived from NPC samples, some variation is possible (Supplementary Figs. S1, S2).

**Figure 10:**
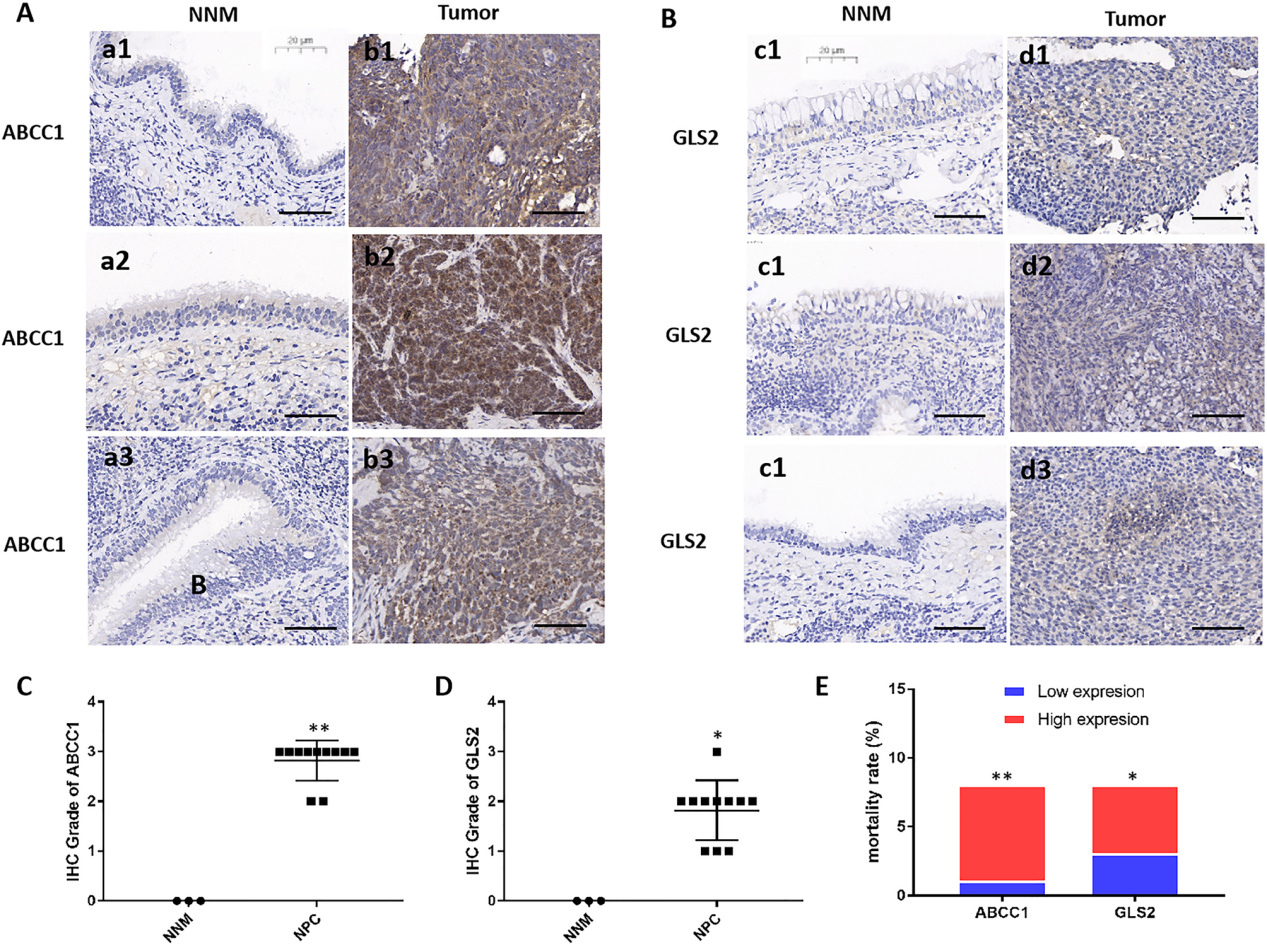
The expression and prognosis of ABCC1 and GLS2 in NPC tissues. (**A,B**) The expression of ABCC1 and GLS2 in 3 nasopharyngeal carcinomas and adjacent non-tumor tissues by IHC. The scale bar was 20 μm. (**C,D**) Statistical analysis to assess the expression level of ABCC1 and GLS2 in NPC tissues. (**E**) Mortality rate testing assessed the correlation between ABCC1 and GLS2 low/high expression with the poor survival of the patients. *p < 0.05, **p < 0.01.

To verify the potential effect of ABCC1 and GLS2 expression in NPC, IHC was performed to analyze ABCC1 and GLS2 expression in NPC tissues (Fig. 10A,B), and the clinical information related to 11 samples, such as sex, age, histological type, and fustat (Supplementary Table S1), was assessed. Compared with normal nasopharyngeal mucosa (NNM), the expression of ABCC1 and GLS2 was upregulated in NPC tissues (*p < 0.05; **p < 0.01) (Fig. 10C,D). This result indicated that ABCC1 and GLS2 were overexpressed in NPC. In addition, we investigated the relationship between high/low expression of ABCC1 and GLS2 and poor survival in tissues. The results showed that the patients with high expression of ABCC1 and GLS2 had a higher mortality rate than those with low expression of ABCC1 and GLS2, which indicated that a high expression of ABCC1 and GLS2 was related to poor survival, as shown in Fig. 10E.

## Discussion

Iron dependency is a characteristic of cancer cells; excessive ionic iron causes “iron enrichment” and leads to ferroptosis, which is a new cell death mode characterized by GPX4 inactivation and ROS accumulation^23^. Ferroptosis is involved in multiple biological processes, including degenerative diseases, carcinoma and T-cell immunity. With the continuous progress of basic and clinical research, ferroptosis has been applied to anticancer therapies, including chemotherapy, radiotherapy and immunotherapy^24,25^. Tumor-based ferroptosis may be a potential biomarker of cancer immunotherapy^26,27^. Consequently, exploring the role of ferroptosis is vital to enhancing the efficacy of cancer therapy.

This study identified four FRGs, ABCC1, GLS2, CS and HMGCR, in NPC. ABCC1 was associated with increased aggressiveness and poor survival in various tumors^28–30^. ABCC1 has been shown to be overexpressed in breast carcinoma and was correlated with poor prognosis and chemotherapeutic sensitivity^31,32^. Moreover, ABCC1 is associated with an increased risk of tumor progression, treatment resistance or death in CHL patients^12^. Our study revealed that ABCC1 was upregulated and correlated with prognosis. These results indicate that the ferroptosis-related gene ABCC1 is a risk factor and may be a promising therapeutic target for NPC. GLS2 is a mitochondrial glutaminase. It catabolizes glutamine to glutamate, which plays a vital role in cancer progression^33^. As a direct target gene of p53, GLS2 is associated with ferroptosis^34^. Previous studies suggested that GLS2 may play different roles depending on tumor type^35,36^. A few studies have suggested that GLS2 expression is associated with good prognosis; for example, high expression of GLS2 in HCC is associated with a long survival time of patients^37^. However, most studies have shown that overexpression or amplification of GLS2 was associated with poor survival prognosis, such as in breast cancer^38,39^, glioblastoma^40^, ovarian cancer^41^, and lung cancer^34,42^. In addition, GLS2 was more highly expressed and regulated by METTL3 by m6A modification, which was significantly associated with poor prognosis in esophageal squamous cell carcinoma^43^. These results are consistent with our results, and further studies are needed to confirm the molecular mechanisms of GLS2 in NPC. In this study, two FRGs (ABCC1 and GLS2) were used in the prognostic risk model, which was an independent prognostic biomarker related to EB subtype, clinical stage and survival status in NPC.

The tumor microenvironment (TME) is a unique environment with infiltrating immune and nonimmune cells^44^. Immune components can modulate the occurrence and development of cancers in the TME^45–47^. In this study, functional enrichment analyses demonstrated that the risk model was involved in immune-based pathways. Immune-infiltrating cells (T follicular helper cells, resting DCs) were markedly upregulated, while immune-infiltrating cells (naive B cells, memory B cells) were downregulated in the high-risk group. Consistent with previous studies, B-cell and memory B-cell expression was markedly lower than that in noncancerous tissues and was correlated with PFS in NPC^48^. These results revealed that FRG-based risk score models were linked to the prognosis of NPC, perhaps partly by regulating the TME.

Proper selection of the correct medications may increase the effectiveness of anticancer drugs for treated patients^49^. The correct diagnosis is critical to determining the correct treatment^50^. To explore the potential role of ferroptosis in drug therapy, our results suggested that the high-risk group was sensitive to some chemotherapy drugs, including docetaxel, embelin, and epothilone B, parthenolide, thapsigargin, tipifarnib, axitinib, and vinorelbine, indicating that these chemotherapy drugs may benefit high-risk NPC patients.

In this study, we analyzed the prognostic role of ferroptosis-related genes in NPC. Two FRG signatures were established to predict overall survival and immunotherapy in NPC patients. In conclusion, these results revealed a ferroptosis-related signature as a potential biomarker in NPC and provided deeper insights into the prognosis and therapeutic targets of NPC.

## Materials and methods

### Analyzing the differential expression of FRGs

The raw data for NPC patients were searched in GEO (https://www.ncbi.nlm.nih.gov/geo/). Differentially expressed genes (DEGs) were obtained with |log2FC|> 1 and an adjusted p < 0.05 between NPC tissue and control tissue from GSE53819 and GSE12452 using the "edgeR" package (version 4.0.5, https://cran.r-project.org/)^51^.

### Estimation of consensus clustering

Consensus clustering (the R package “ConsensusClusterPlus”, https://cran.r-project.org/) was used to analyze four prognosis-related ferroptosis genes (ABCC1, HMGCR, CS and GLS2), and k = 3 may be the best choice in the GSE102349 datasets. Kaplan‒Meier (K-M) survival curves were used to calculate the prognostic signature. The correlation analysis of clustering and risk classification was performed using the R packages “ggalluvial” (version 0.12.2, https://cran.r-project.org/src/contrib/Archive/ggalluvial/) and “ggplot2” (version 3.0.0, https://cran.r-project.org/src/contrib/Archive/ggplot2/).

### Investigation and prediction of prognostic signatures

Univariate Cox regression investigated the PFS from the GSE102349 dataset of NPC and four ferroptosis genes (ABCC1, HMGCR, CS, GLS2), for which hazard ratios (HRs) were calculated. LASSO regression was used to calculate the risk score using the R package “glmnet” (version 4.0, https://cran.r-project.org/src/contrib/Archive/glmnet/). ROC curve was used to evaluate the prognostic model using the R package “survivalROC” (version 1.0.2, https://cran.r-project.org/src/contrib/Archive/survivalROC/). Univariate/multivariate PFS analyses assessed the prognostic score. The nomogram was used to integrate poor progression-free survival using the R package “rms” (version 6.0, https://cran.r-project.org/src/contrib/Archive/rms/). p < 0.05 indicates statistical significance.

### Identifying immune infiltration

GO and KEGG analyses (https://www.kegg.jp/kegg/kegg1.html) were used for the enrichment of DEGs obtained with |log2FC|> 1 and adjusted p < 0.05 using the R packages “clusterProfiler” and “enrichplot”^52^. CIBERSORT^53^ (https://cibersort.stanford.edu/) was used to detect the distribution of immune cells in GSE102349 (containing 113 NPC patients, 54 of whom had survival data)^54^, this data set (GSE102349) contains annotations about the distribution of intratumoral and stromal TILs.

### Predicting the drug response of patients with high/low risk

The half-maximal inhibitory concentration (IC50) values of compounds/inhibitors for high/low risk nasopharyngeal carcinoma patients were predicted using the R “pRRophetic” package as previously described^55^.

### Immunohistochemistry

Eleven nasopharyngeal carcinoma and three NNM collected from the Second Xiangya Hospital of Central South University from January 2018 to December 2018 were examined and included in this study, and the ethics committee approved this study. The patients obtained informed consent. The following primary antibodies were used: rabbit polyclonal anti-ABCC1 (AF7503, 1:500, Beyotime, China) and anti-GLS2 (bs-13376R, 1:500, Bioss, China). The secondary antibody was HRP-conjugated goat anti-rabbit (ab97051, 1:500, Sigma, USA). Sections were photographed by a microscope. The scores of staining intensity of tumor cells were as follows: 0 (no coloring); 1 (≤ 30%); 2 (31–60%); and 3 (61–100%). IHC was performed as previously described^56^.

### Statistics

SPSS software 20.0 (SPSS, Inc., USA) and GraphPad Prism 7.0 (La Jolla, CA, USA) were used to analyze the data. p < 0.05 indicates statistical significance.

### Ethics statement

The studies involving human participants were reviewed and approved by the ethics committee of the Second Xiangya Hospital, Central South University. The patients/participants provided their written informed consent to participate in this study. All methods were carried out in accordance with relevant guidelines and regulations.

## Supplementary Information

Supplementary Information.

## Data Availability

Publicly available datasets were analyzed in this study. This data can be found here: The raw data and corresponding clinical information were downloaded from Gene Expression Omnibus Database (GEO; https://www.ncbi.nlm.nih.gov/geo/). The expression array data GSE53819 (https://www.ncbi.nlm.nih.gov/geo/query/acc.cgi?acc=GSE53819) containing 18 control tissue and 18 NPC tissue and the throughput sequencing data GSE12452 (https://www.ncbi.nlm.nih.gov/geo/query/acc.cgi?acc=GSE12452) containing 10 control tissue and 31 NPC tissue and the GSE102349 (https://www.ncbi.nlm.nih.gov/geo/query/acc.cgi?acc=GSE102349) dataset containing 113 NPC patients used for subsequent analysis. All supporting data are included within the article, and all the data generated in this article are available from the first author on reasonable request.
