# Supplementary material for: The ferroptosis signature predicts the prognosis and immune microenvironment of nasopharyngeal carcinoma
Source: Sci Rep. 2023 Feb 2;13:1861. doi: 10.1038/s41598-023-28897-2 (PMC9895067; doi:10.1038/s41598-023-28897-2)
Supplement: Supplementary file 1 — Supplementary Information. [file 41598_2023_28897_MOESM1_ESM.docx]

Table S1 | Correlation between ABCC1 and GLS2 expression and clinicopathological characteristics in nasopharyngeal carcinomas(n=11) .

|  |  | ABCC1 | | GLS2 | |
| --- | --- | --- | --- | --- | --- |
|  |  | Low expresion | High expresion | Low expresion | High expresion |
| Gender |  |  |  |  |  |
| Male | 8 | 1 | 7 | 3 | 5 |
| Female | 3 | 1 | 2 | 2 | 1 |
| Age |  |  |  |  |  |
| ≤55 | 3 | 1 | 2 | 0 | 3 |
| >55 | 8 | 1 | 8 | 3 | 6 |
| Histologic type (WHO) |  |  |  |  |  |
| Differentiation | 5 | 1 | 4 | 1 | 4 |
| Undifferentiation | 6 | 1 | 5 | 2 | 4 |
| Fustat |  |  |  |  |  |
| Alive | 3 | 1 | 2 | 0 | 3 |
| Dead | 8 | 1 | 7 | 3 | 5 |


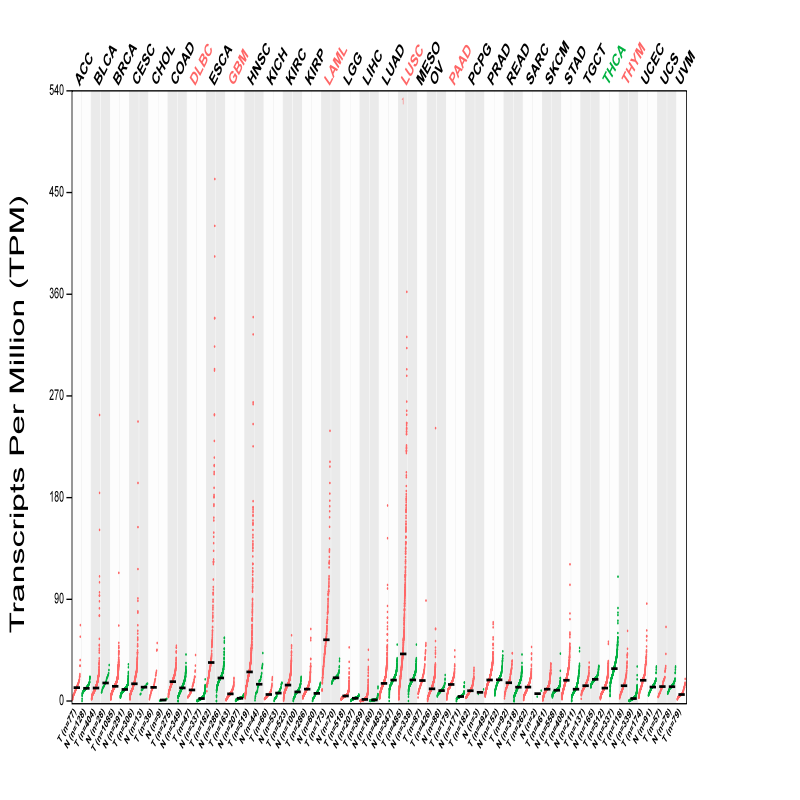


Figure S1. The expression of ABCC1 in pan-cancer using GEPIA2


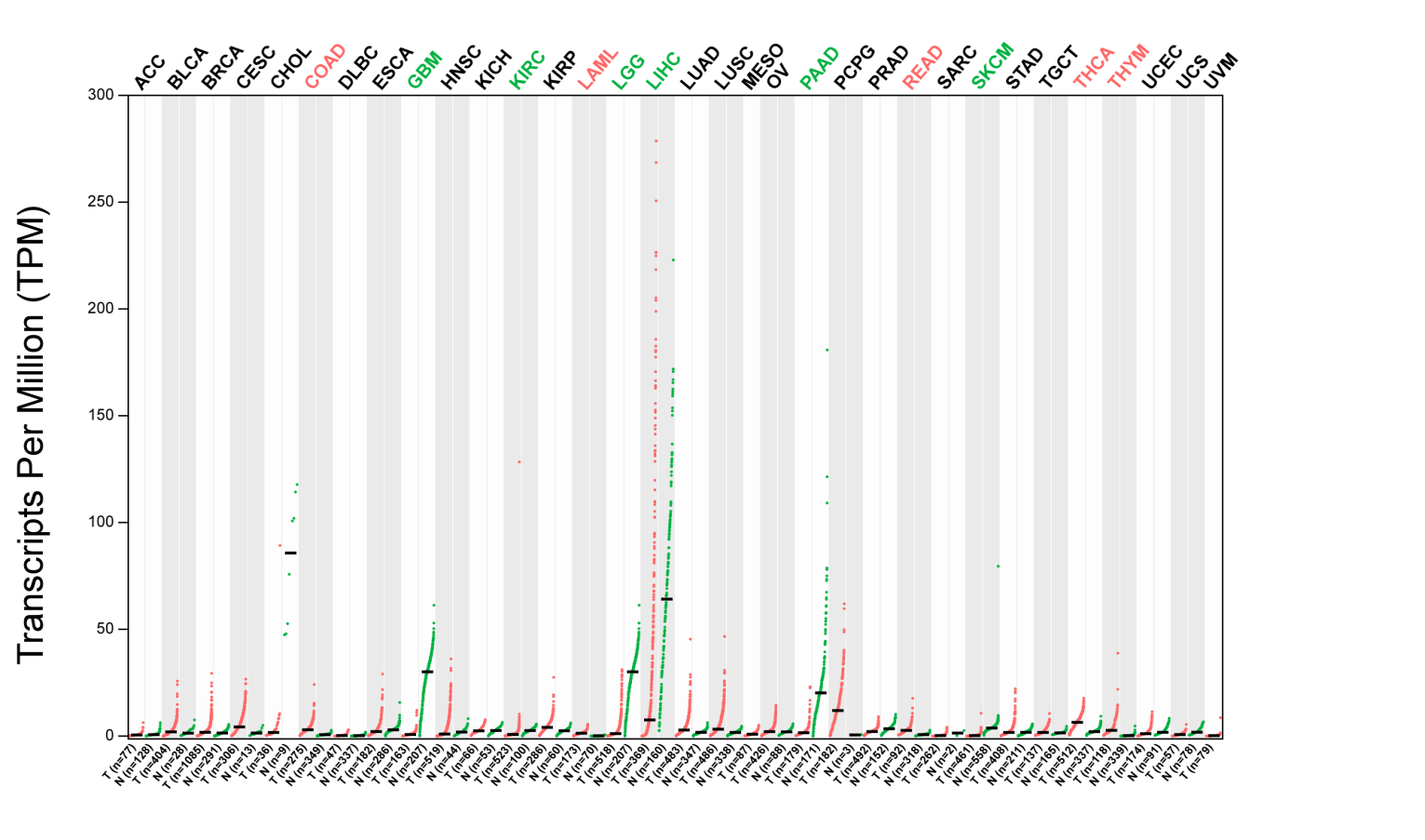


Figure S2. The expression of GLS2 in pan-cancer using GEPIA2
